# Fas/Fas ligand regulation mediates cell death in human Ewing's sarcoma cells treated with melatonin

**DOI:** 10.1038/bjc.2012.66

**Published:** 2012-03-01

**Authors:** G García-Santos, V Martin, J Rodríguez-Blanco, F Herrera, S Casado-Zapico, A M Sánchez-Sánchez, I Antolín, C Rodríguez

**Affiliations:** 1 Departamento de Morfología y Biología Celular, Facultad de Medicina, Universidad de Oviedo, C/ Julian Claveria 6, 33006 Oviedo, Spain; 2 Instituto Universitario de Oncología del Principado de Asturias (IUOPA), Oviedo, Spain; 3 Instituto de Medicina Molecular, Faculdade de Medicina da Universidade de Lisboa. Avenida Professor Egas Moniz, 1649-028 Lisboa, Portugal

**Keywords:** apoptosis, ESFT cell lines, Fas/Fas L upregulation, melatonin

## Abstract

**Background:**

Despite recent advances in cancer therapy, the 5-year survival rate for Ewing's sarcoma is still very low, and new therapeutic approaches are necessary. It was found previously that melatonin induces cell death in the Ewing's sarcoma cell line, SK-N-MC, by activating the extrinsic apoptotic pathway.

**Methods:**

Melatonin actions were analysed by metabolic viability/survival cell assays, flow cytometry, quantitative PCR for mRNA expression, western blot for protein activation/expression and electrophoretic mobility shift assay for transcription factor activation.

**Results:**

Melatonin increases the expression of Fas and its ligand Fas L, this increase being responsible for cell death induced by the indolamine. Melatonin also produces a transient increase in intracellular oxidants and activation of the redox-regulated transcription factor Nuclear factor-kappaB. Inhibition of such activation prevents cell death and Fas/Fas L upregulation. Cytotoxic effect and Fas/Fas L regulation occur in all Ewing's cell lines studied, and do not occur in the other tumour cell lines studied where melatonin does not induce cell death.

**Conclusion:**

Our data offers new insights in the study of alternative therapeutic strategies in the treatment of Ewing's sarcoma. Further attention deserves to be given to the differences in the cellular biology of sensitive tumours that could explain the cytotoxic effect of melatonin and the increase in the level of free radicals caused by this molecule, in particular cancer types.

The Ewing's sarcoma family of tumours (ESFT) are aggressive neuroectodermal neoplasms of bone and soft tissues, with a peak incidence during childhood and adolescence. These types of tumours are characterised by the presence of a chromosomal translocation that produces a chimeric protein. Approximately 85% of these tumours carry the translocation t(11;22)(q24;q12), which produces the fusion protein EWS-FLI1 (Ewing's sarcoma type I) (de Alava and Gerald, 2000), although other less common translocations have been described (mostly Ewing's sarcoma types II and III). The fusion protein acts as an aberrant transcription factor and has the ability to modulate the transcription of specific genes involved in oncogenesis (Uren and Toretsky, 2005). Despite recent advances in cancer therapy, the 5-year survival rate for ESFT is still very low (Damron *et al*, 2007), and new therapeutic approaches are necessary.

The natural indolamine melatonin (*N*-acetyl-5-methoxytryptamine) has previously been shown to possess antitumoural properties that are generally mediated by the inhibition of tumour cell proliferation (Hill and Blask, 1988; Sainz *et al*, 2003; Reiter, 2004; Martin *et al*, 2006). There are, however, a few reports that show a cytotoxic effect of melatonin in some types of cancer cells (Trubiani *et al*, 2005; Buyukavci *et al*, 2006; Rubio *et al*, 2007; Martin-Renedo *et al*, 2008). Also, some authors found an improvement in chemotherapeutic regimes when melatonin treatment was included (Lissoni *et al*, 1999; Martin *et al*, 2010). Moreover, there are no publications reporting that melatonin could kill normal cells. Quite on the contrary, melatonin typically protects normal cells from a variety of insults (Antolín *et al*, 2002; Reiter *et al*, 2003; Bruck *et al*, 2004; Herrera *et al*, 2007).

We had previously demonstrated that melatonin induces apoptotic cell death in the Ewing's sarcoma cell line, SK-N-MC, (Garcia-Santos *et al*, 2006) and presents synergism with vincristine and ifosfamide when administered to Ewing's cells in combination with such chemotherapeutic drugs (Casado-Zapico *et al*, 2010). The said synergism is the result of the potentiation of the extrinsic pathway of apoptosis. Combination of melatonin plus vincristine or ifosfamide potentiated the caspase 8 activation that is induced by each drug given alone (Casado-Zapico *et al*, 2010). The extrinsic pathway of apoptosis is activated after binding of death receptors to their ligands, followed by trimerisation of receptors and afterwards by recruitment of adaptor proteins. Such proteins in turn recruit caspase 8 to form the death-inducing signalling complex. The conversion of pro-caspase 8 to active caspase 8 by cleavage occurs at this membrane-bound complex. Active caspase 8 can cleave executioner caspases with scission of multiple targets that determine the typical features of apoptosis (Fulda and Debatin, 2006).

The next logical question on the cytotoxic effect of melatonin in Ewing's sarcoma cells referred to the intracellular signalling pathways involved in the induction of such apoptosis by this indolamine. Besides the previous results with melatonin administration mentioned above, we also had previously found that this indolamine sensitises glioma cells to TRAIL treatment, probably due to the increase in the death receptor DR5 expression (Martin *et al*, 2010). On the other hand, there are reports of sensitivity to TRAIL of Ewing's sarcoma cell lines (Kumar *et al*, 2001; Picarda *et al*, 2010). All these data together suggested the hypothesis that melatonin could be regulating the expression of a death receptor and /or its ligand. We demonstrate that this molecule increases the expression of Fas and its ligand Fas L. We also demonstrate that this increase is responsible for cell death induced by the indolamine in Ewing's sarcoma cells and that this cytotoxic effect occurs in all Ewing's cell lines studied. Increment of Fas/Fas L expression does not occur in other tumour cell lines where melatonin does not induce cell death.

## Materials and methods

### Cell culture and reagents

Culture flasks and dishes were obtained from Falcon (Becton Dickinson BioScience, Le Pont de Claix, France). Ewing's sarcoma SK-N-MC cell line (EWS-FLI1 type 1) and neuroblastoma cell lines SK-N-SH, SK-N-SY5Y and SK-N-AS were purchased from the European Collection of Cell Cultures (Salisbury, United Kingdom). Ewing's sarcoma cell lines TC-71 and A673 (type 1), SK-ES1 (type 2) A4573 (type 3) were a generous gift from Dr JA Toretsky (Departments of Oncology and Pediatrics, Georgetown University, Washington DC, USA). The presence of the Ewing's sarcoma-specific transcription factor EWS-FLI1 (type 1 for SK-N-MC, TC-71 and A673; type 2 for SK-ES1; type 3 for A4573) was determined by PCR in these cell lines to confirm their origin. Caspase-8 inhibitor Z-IETD-FMK was purchased from Calbiochem (La Jolla, CA, USA). Anti-FAS antibody (clone ZB4) and anti-FAS-L antibody (clone NOK1) used in the neutralisation assays were obtained from Millipore (Billerica, MA, USA) and BD Pharmingen (Franklin Lakes, NJ, USA), respectively, and added to the cellular medium 4 h before treatment with melatonin. All other reagents were purchased from Sigma (Sigma-Aldrich, Milwaukee, WI, USA), unless otherwise indicated.

### Cell viability assays

For the MTT assay, cells were plated in 96-well dishes. Assays were carried out as described by Casado-Zapico *et al* (2010). Samples were measured in an automatic microplate reader (*μ*Quant, Bio-Tek Instruments, Inc., Winooski, VT, USA) at the wavelength of 540 nm.

For the lactate dehydrogenase release assay, cells were seeded in 24-well plates. After treatment with melatonin or vehicle for the indicated time, determination of total and released LDH activity was accomplished following specifications of the *In Vitro* Toxicology assay kit (Sigma). Absorbance was determined using an automatic microplate reader (*μ*Quant; Bio-Tek Instruments, Inc.) at 490 nm.

### Caspase-3 activity

After treatment, activation of caspase-3 was determined using the fluorometric caspase-3 assay kit (Sigma Chemical Co., St Louis, MO, USA), following the recommendations of the manufacturer. After 2-hr incubation of the reaction mixture at room temperature in darkness, samples were analysed in a microplate fluorimeter FLX-800 (Bio-Tek Instruments, Inc.) at an excitation wavelength of 360 nm and an emission wavelength of 460 nm.

### Flow cytometry analysis of intracellular peroxides

Intracellular production of peroxides was evaluated by using the fluorescent probe 6-carboxy-2′,7′-dichlorodihydrofluorescein diacetate as described by Casado-Zapico *et al* (2010). The DCF fluorescence of 10 000 live cells per group was measured in a Beckman Coulter FC500 flow cytometer (Becton Dickinson).

### Electrophoretic mobility shift assay (EMSA)

To determine nuclear factor kappaB (NF-*κ*B) activation, EMSA was carried out. Nuclear extracts were obtained following the method described by Dignam *et al* (1983). Oligonucleotide probes containing the consensus sequence for NF-*κ*B (Santa Cruz Biotechnology, Santa Cruz, CA, USA) were labelled with (*α*-32P)ATP (3000 Ci mmol^−1^) using T4 polynucleotide kinase 5′-end labelling kit (Amersham Life Science, Pittsburgh, PA, USA). A 10-*μ*g per sample of nuclear extracts were incubated on ice for 30 min, with 0.4 ng of the labelled oligonucleotide. The specificity of binding was determined by competition with the unlabelled oligonucleotide (100-fold excess). Protein–DNA complexes were resolved on 6% non-denaturing polyacrylamide gels at 250 V for 1.5 h in TBE. Gels were dried and exposed to Kodak Biomax *X* ray film (Amersham Life Science).

The optical density (O.D.) of the bands was estimated using an HP scanjet 3670 scanner (HP, Madrid, Spain) and the Scion Image Alpha 4.0.3.2 free analysis software (Scion Corp., Frederick, MD, USA).

### Western blot

After treatments, western blot analyses were done using standard methods (Casado-Zapico *et al*, 2010). Blots were incubated overnight at 4 °C with appropriate antibodies: anti-Bcl-2, anti-Bcl-Xl, anti-Bcl-xS, anti-Bax, anti-Bak and anti-caspase-8, which recognises the full and cleavage forms of the protein (Calbiochem), anti-Bid, anti-caspase-9, which also recognises the full and cleavage forms, and anti-FAS-L (Cell Signaling Technology, Beverly, MA, USA), anti-GAPDH and anti-FAS (Santa Cruz Biotechnology). Immunoreactive polypeptide was visualised using horseradish peroxidase-conjugated secondary antibodies anti-mouse and anti-rabbit (Calbiochem) and chemiluminescence detection reagents (Amersham Life Science), following the procedures supplied by the manufacturers. The O.D. of the bands was estimated as described above for EMSA.

### Quantitative reverse transcription PCR (qRT–PCR)

The RNA extraction, reverse transcription and quantitative analysis of mRNA levels were carried out as described by Martin *et al* (2010). The sequences of the sense and antisense primers for human TNFR1, TNF-*α*, FAS-R, FAS-L, DR4, DR5, TRAIL and GADPH (housekeeping gene for normalisation) are described in Table 1. Each sample was tested in triplicate, and analyses of relative gene expression data were done using the 2−ΔCT method.

### Flow cytometric analysis of FAS and FAS-L expression

For cell surface FAS or FAS-L protein measurement, tumour cells were stained with anti-FAS antibody (clone ZB4) and anti-FAS-L antibody (clone NOK1) (Billerica, MA, USA) or isotype-matched control for 1 h at 4 °C, washed three times and then incubated with PE-conjugated secondary antibody (Invitrogen, Grand Island, NY, USA) for another 30 min. Fluorescence of 10 000 cells in each experimental group were then analysed by flow cytometry in a Beckman Coulter FC500 flow cytometer (Becton Dickinson).

### Data analysis

Results are the average value of at least three independent experiments. Data are represented as the mean±s.e.m. Significance was tested by one-way ANOVA followed by a Student–Newman–Keuls multiple range test. Statistical significance was accepted when *P*⩽0.05.

## Results

### Treatment with melatonin of the Ewing's sarcoma cell line SK-N-MC induces an increase in Fas and Fas L expression that is responsible for cell death induction

**Figure 1:**
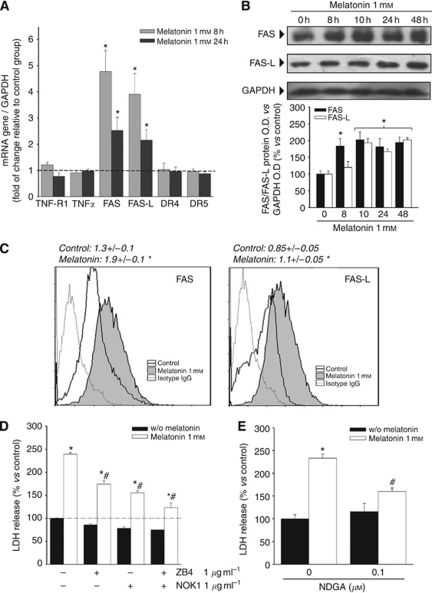
Fas/Fas L expression after treatment with melatonin. (**A**) SK-N-MC cells were treated with or without 1 mM melatonin for 8 or 24 h, and TNFR1, TNF-*α*, Fas, Fas-L, DR4, DR5 and TRAIL expression were determined by quantitative PCR to assess mRNA expression levels. GAPDH was used as a housekeeping gene. Relative gene expressions are represented as the *n*-fold increase compared with basal level (vehicle-treated cells: dotted line). ^*^P<0.05 *vs* vehicle-treated cells. (**B**) Expression of Fas and Fas L proteins was determined by western blot after treatment with 1 mM melatonin for the indicated times. GAPDH was used as housekeeping gene. A representative blot is shown and densitometric analysis of the immunoblots of three independent experiments is represented below. ^*^P<0.05 *vs* vehicle-treated group (0 h). (**C**) Flow cytometric analysis (FASCS) of SK-N-MC cells treated with 1 mM melatonin for 48 h. Cells were stained with anti-FAS (left panel) or anti-FAS-L (right panel) and then PE-conjugated secondary antibody. The values obtained are shown above the graphs. ^*^P<0.05 *vs* control (vehicle-treated group). (**D**) Overexpression of FAS/FAS-L mediates melatonin cytotoxic effect. SK-N-MC cells were incubated with 1 *μ*g ml^−1^ of neutralising antibody against FAS (ZB4), FAS-L (NOK1) or both, 4 h prior to the addition of melatonin 1 mM. Cell death was evaluated by the release of lactate dehydrogenase (LDH) after 72 h. ^*^P<0.05 *vs* vehicle-treated cells; ^#^*P*<0.05 *vs* melatonin 1 mM-treated group. (**E**) Evaluation of cell death by the release of LDH after 72 h of incubation with melatonin plus 0.1 *μ*M of nordihydroguaiaretic acid (NDGA); ^*^P<0.05 *vs* vehicle-treated group; ^#^*P*<0.05 *vs* melatonin 1 mM-treated group.

Given that the first step usually required for activation of the extrinsic pathway of apoptosis is the binding to death receptors of their ligands, we first studied the expression of the death receptors TNFR1, Fas, DR4 and DR5, and their respective ligands TNF-*α*, Fas L and TRAIL by qRT–PCR, after melatonin treatment of SK-N-MC cells. Incubation with this indolamine increased the expression of Fas and Fas L. We found a 4.5-fold increase for Fas and almost 4-fold for Fas L after 8 h of treatment when compared with non-treated cells. The amount of mRNA was twice the value found in non-treated cells after 24 h. No increase in any other death receptor or ligand was found (Figure 1A). No signal was detected for TRAIL (data not shown).

The expression of Fas and Fas L was subsequently studied by western blot. Both proteins showed a 2-fold increase with respect to non-treated cells. The increase was first observed after 8 h of treatment in the case of Fas and after 10 h in the case of Fas L, and remained for at least during 48 h (Figure 1B). These results were confirmed by the study of the amount of Fas and Fas L bound to the cell surface by flow cytometry (PE fluorescence), showing an increase in bound protein in both cases (Figure 1C).

To ascertain whether the increase in Fas and Fas L expression was relevant in the cell death induced by melatonin, SK-N-MC cells were co-incubated with the indolamine plus the Fas antagonist ZB4 or the Fas L antagonist NOK1, or with melatonin plus both antagonists at the same time. Both Fas and Fas L antagonists partially prevented the induction of cell death by melatonin (50% and 60%, respectively), whereas co-incubation with both compounds simultaneously reduced the cell death induced by the indolamine by >80% (Figure 1D). Treatment with a lipooxigenase inhibitor, associated with the prevention of cell death induced by Fas, NDGA, also partially prevented cell death induced by melatonin by >50% (Figure 1E).

Finally, we wanted to confirm that all these events are really related to the activation of the extrinsic pathway induced by melatonin, and to study the possible contribution of the intrinsic pathway of apoptosis. A time-course study of caspase 8 and 9 activation as well as the expression of proteins related to the intrinsic pathway of apoptosis were achieved by western blot. We also analysed Bid, the protein responsible for the activation of the intrinsic pathway after caspase-8 activation.

**Figure 2:**
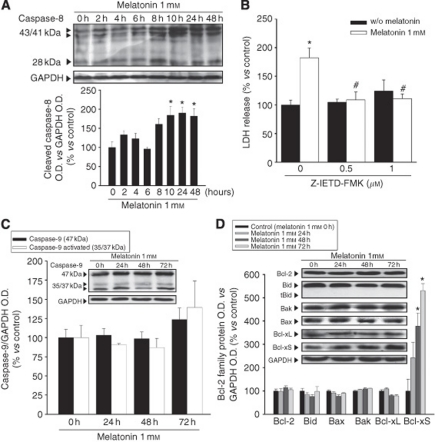
Melatonin cytotoxic effect is dependent only on the extrinsic pathway of apoptosis. (**A**) Western blot analysis of caspase-8 activation. SK-N-MC cells were incubated with 1 mM melatonin at the indicated times. Signals seen at 43/41 and 28 kDa molecular weights correspond to cleaved forms of caspase-8. Histogram presents the densitometric analysis from at least three independent experiments; ^*^P<0.05 *vs* vehicle-treated group (0 h). (**B**) Evaluation of cell death – as LDH release – after 72 h of treatment with melatonin plus the indicated concentrations of caspase-8 inhibitor Z-IETD-FMK. ^*^P<0.05 *vs* vehicle-treated cells, ^#^*P*<0.05 *vs* melatonin 1 mM group. (**C**) Western blot analysis of caspase-9 activation and (**D**) western blot analysis of Bcl-2 family protein expression. SK-N-MC cells were incubated with 1 mM melatonin for the indicated times. Histograms represent densitometric analysis from at least three independent experiments; ^*^P<0.05 *vs* vehicle-treated group (0 h).

Activation of caspase-8 was found after 10 h of treatment and was sustained for at least until 48 h after treatment (Figure 2A). Treatment with the caspase-8 inhibitor Z-IETD-FMK totally prevented cell death induced by melatonin (Figure 2B). No activation of caspase-9 was found (Figure 2C). The Bcl-2 family of proteins studied did not show variation either. Only Bcl-Xs, a pro-apoptotic splicing variant of Bcl-X, increased significantly after 48 h of melatonin treatment (Figure 2D).

### Increase in intracellular oxidants after melatonin treatment is involved in the cell death induced by this molecule

**Figure 3:**
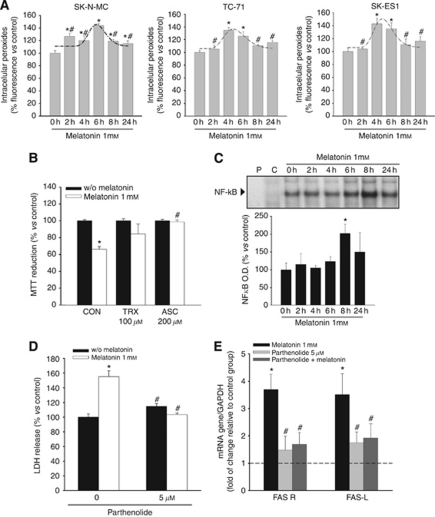
ROS signalling involvement in melatonin cytotoxic effect. (**A**) Ewing's sarcoma cells were treated with melatonin 1 mM at the indicated times. Intracellular peroxides were measured by flow cytometry and expressed as % fluorescence *vs* vehicle-treated group (0 h); ^*^P<0.05 *vs* vehicle-treated cells; ^#^*P*<0.05 *vs* group with highest value. (**B**) SK-N-MC cells were incubated with or without melatonin 1 mM, Trolox (TRX) 100 *μ*M, ascorbic acid (ASC) 200 *μ*M or the combination for 72 h. Cell viability was determined by the MTT reduction assay and cell number after the combined treatments was expressed as % *vs* each single treatment group; ^*^P<0.05 *vs* each single treatment group, ^#^*P*<0.05 *vs* melatonin 1 mM group. (**C**) Melatonin activates NF-*κ*B pathway. NF-*κ*B DNA-binding ability analysed by EMSA. SK-N-MC cells were incubated with 1 mM melatonin at the indicated times: (P), labelled probe; (C), control for specificity (100-fold excess of unlabelled probe). Histogram represents densitometric analysis from at least three independent experiments; ^*^P<0.05 *vs* vehicle-treated group (0 h). (**D**) The inhibitor of NF-*κ*B activation parthenolide prevents cell death induced by melatonin 1 mM. Cells were treated for 72 h with melatonin with or without 5 *μ*M parthenolide, and cell death was evaluated as the LDH release. ^*^P<0.05 *vs* vehicle-treated cells, ^#^*P*<0.05 *vs* melatonin 1 mM group. (**E**) The inhibitor of NF-*κ*B activation parthenolide prevents upregulation of Fas and Fas L induced by melatonin 1 mM. Cells were treated with melatonin, with 5 *μ*M parthenolide or with their combination, and Fas and Fas L expression was evaluated by quantitative PCR. GAPDH was used as a housekeeping gene. Relative gene expressions are represented as the *n*-fold increase compared with basal level (dotted lines represent vehicle-treated cells). ^*^P⩽0.05 *vs* vehicle-treated cells. ^#^*P*<0.05 *vs* melatonin 1 mM group.

It has been previously shown that death receptor activation increases intracellular oxidants (Devadas *et al*, 2003; Sato *et al*, 2004). There are also some reports on the pro-oxidant effects of melatonin in a few human tumour cell lines where it induces cell death (Osseni *et al*, 2000; Wolfler *et al*, 2001). We, therefore, analysed the possible involvement of ROS in melatonin-induced apoptosis in Ewing's sarcoma cells. We found that melatonin significantly increases intracellular ROS production in SK-N-MC, TC-71 and SK-ES1 (Figure 3A). Production of ROS is increased after 2 h of incubation with 1 mM melatonin, reaches a maximum after 4–6 h and falls after 8 h (Figure 3A). The involvement of ROS production in the cytotoxic action of melatonin was confirmed by the use of two well-known antioxidants. We incubated the SK-N-MC cell line with ascorbic acid (200 *μ*M) or trolox (100 *μ*M) in the presence or absence of melatonin 1 mM for 72 h, and found that both trolox and ascorbic acid prevent the cytotoxicity of melatonin (Figure 3B).

### Activation of the transcription factor NF-kappaB is responsible for cell death and Fas/Fas L increase after the treatment with melatonin

Endogenous free radicals are involved in the induction and maintenance of signal transduction pathways regulating, among other processes, cell proliferation and cell death. One of the proteins directly regulated by the intracellular redox state is the transcription factor NF-*κ*B, which is activated by oxidation and inactivated by reduction (Schoonbroodt and Piette, 2000) and has been shown to be regulated by melatonin in other experimental systems (Chuang *et al*, 1996; Post *et al*, 1998; Bruck *et al*, 2004). NF-*κ*B activation has been shown indeed to be critical in the signalling pathway leading to Fas expression induced by TNF-*α* (Starace *et al*, 2005), and its role in the activation of Fas promoter has been previously reported (Hayden and Ghosh, 2004). It is also involved in the upregulation of Fas expression after the treatment with interleukin-12 in Ewing's sarcoma cells (Chan *et al*, 1999). We found that melatonin increases the activation of NF-*κ*B in SK-N-MC cells, reaching a peak after 8 h of incubation and decreasing thereafter (Figure 3C). Co-incubation with parthenolide, a well-known inhibitor of NF*κ*B activation, prevented both cell death (Figure 3D) and increase in Fas and Fas L expression (Figure 3E) induced by melatonin.

### Effect on Fas and Fas L upregulation after melatonin treatment also occurs in other Ewing's sarcoma cell lines but not in other cancer cell types that are non-sensitive to the cytotoxic effect of this molecule

**Figure 4:**
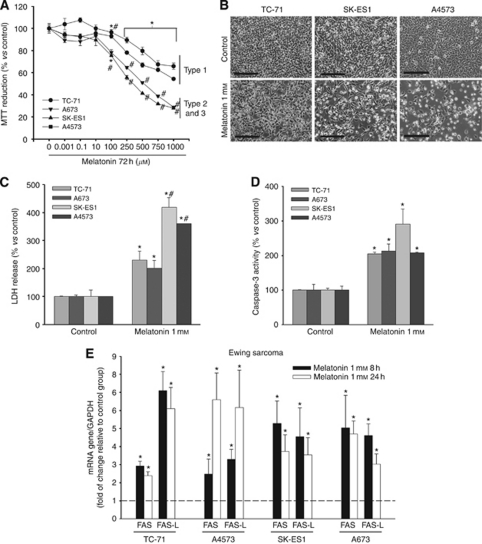
Melatonin specifically induces cell death in a panel of Ewing's sarcoma cell lines. (**A**) TC-71, A673 (EWS-FLI1 type 1), SK-ES1 (EWS-FLI1 type 2) and A4573 (EWS-FLI1 type 3) cell number, determined by MTT assay decreased after 72 h of treatment with melatonin in a dose-dependent manner; ^*^P<0.05 *vs* vehicle-treated group. There are also statistical differences in the effect of melatonin between the EWS-FLI1 type 1 cells and EWS-FLI1 type 2, 3 cells (^#^*P*<0.05). (**B**) Decrease in the number of cells in Ewing's sarcoma (TC-71, SK-ES1 and A4573). A rise in cellular debris is observed under phase contrast microscopy after 72 h of melatonin treatment. Bars: 50 *μ*m. (**C**), Increase of cell death in Ewing's cells was evaluated by the release of LDH into the extracellular medium, after 72 h of incubation with melatonin; ^*^P<0.05 *vs* vehicle-treated cells; ^#^*P*<0.05 *vs* EWS-FLI1 type 1 cells. (**D**) Increase in caspase-3 activity in a panel of Ewing's cell lines determined after 48 h of incubation with melatonin; ^*^P<0.05 *vs* control groups. (**E**) Fas and Fas L mRNA expression was evaluated in the same cells by quantitative PCR. GAPDH was used as a housekeeping gene. Relative gene expression is represented as the *n*-fold increase compared with basal level (dotted lines represent vehicle-treated cells). ^*^P⩽0.05 *vs* vehicle-treated cells.

The next step was to ensure that cytotocicity and upregulation of Fas and Fas L are general effects of melatonin in Ewing's sarcoma, and not particular effects on SK-N-MC cells. We first studied cell death induction by this indolamine in four other Ewing's sarcoma cell lines (TC-71, A673, -type I-, SK-ES1 –type II- and A4573 –type III). All of them were sensitive to melatonin, showing a decrease in cell viability (Figure 4A), which paralleled an increase in cell death (Figures 4B and C) and caspase 3 activity (Figure 4D). To ensure that Fas and Fas L regulation also took place after melatonin treatment in these cells, we measured mRNAs for both proteins by qRT–PCR, and found upregulation of both of them (Figure 4E). Fas and Fas L increases found in SK-ES1 and A673 were similar to what appeared in SK-N-MC cells; the increases found in TC-71 are higher than in SK-N-MC (a seven-fold increase over the non-treated cells). In the case of A4573 there was an ∼3-fold increase over the control after 8 h of treatment and a markedly higher increase after 24 h of treatment (6- to 7-fold greater than the control).

**Figure 5:**
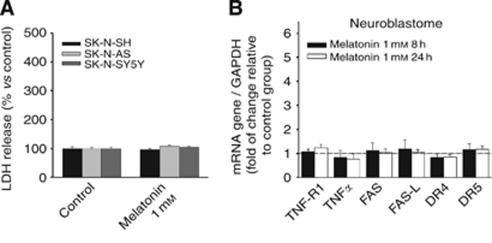
Melatonin treatment neither induces cell death nor death receptor/ligand regulation in neuroblastoma cells. (**A**) Increase in cell death does not occur in neuroblastoma cell lines as evaluated by the release of LDH into the extracellular medium, after 72 h of incubation with 1 mM melatonin. (**B**) SK-N-SH, SK-N-AS and SK-N-SY5Y neuroblastoma cells were treated with or without 1 mM melatonin for 8 or 24 h, and TNFR1, TNF-*α*, Fas, Fas-L, DR4, DR5 and TRAIL expression were determined by quantitative PCR to assess mRNA expression levels. GAPDH was used as a housekeeping gene. Relative gene expressions are represented as the *n*-fold increase compared with basal level (dotted lines represent vehicle-treated cells).

Subsequently, the effect of melatonin on levels of mRNA for death receptors and their ligands was evaluated in one neuroblastoma cell line. Neuroblastoma is especially interesting as it is closely related to Ewing's sarcoma in origin, but it does not present the fusion protein characteristic of the EFST. In this case, melatonin failed to induce cell death (Figure 5A). The fact that there was no variation in mRNAs for Fas and Fas L, and neither for any other death receptors or ligands (Figure 5B) indicates that this indolamine does not regulate such death proteins in a general manner, but it does so very specifically in some tumour cells, where it is able to induce cell death.

## Discussion

Despite the advances made in cancer therapy over the last decade, the 5-year survival rate for Ewing's sarcoma patients remains very low. This emphasises the need for new therapeutic approaches. The main challenge in cancer therapy is to find treatments that specifically target and kill tumoural cells, while being harmless to normally dividing cells. We had previously communicated a pro-apoptotic effect of melatonin in the Ewing's sarcoma cell line SK-N-MC (Garcia-Santos *et al*, 2006) that is mediated by an activation of the extrinsic pathway of apoptosis and which presents a synergistic effect with classical chemotherapeutic drugs such as vincristine or ifosfamide (Casado-Zapico *et al*, 2010). In the present manuscript, we address the expression and functional relevance of the Fas/Fas L system in the cell death induced by melatonin in Ewing's sarcoma. We report the upregulation by melatonin of the death receptor Fas and its ligand Fas L without effect on any other death receptors or ligands. The finding of Fas- and Fas L-neutralising antibodies preventing cell death induced by melatonin is in agreement with the role of such regulation in the cytotoxic effect of this indolamine. Cell death induction and Fas/Fas L upregulation extends to four other Ewing's sarcoma cell lines. Such upregulation does not occur in other non-Ewing's sarcoma cancer cell lines where melatonin does not induce cell death.

The main route for the activation of the extrinsic pathway of apoptosis is the binding to death receptors of their ligands, although some drugs can induce apoptosis through the extrinsic pathway in a Fas L-independent manner (Reis-Sobreiro *et al*, 2009). Such binding is one of the mechanisms used by the host immune system to fight aberrant proliferating tumour cells. In addition, several chemotherapeutic drugs induce cell death in tumour cells by activating this pathway (Friesen *et al*, 1996; Micheau *et al*, 1997; Fulda *et al*, 2000). However, not all tumour cells are sensitive to such activation, and some of them show resistance to this pathway by means of several mechanisms, and therefore manage to evade the host immune system, hence becoming resistant to chemotherapy. Such mechanisms can be, among others, a downregulation of death receptors; a downregulation of caspase-8 expression or an upregulation of inhibitors of apoptosis such as the cellular FLICE inhibitory protein (c-FLIP). The c-FLIP expression by Ewing's sarcoma cells does not interfere with the induction of the extrinsic pathway of apoptosis (Kontny *et al*, 2001; Mitsiades *et al*, 2001). In the present work, we demonstrate that melatonin raises Fas and Fas L expression in Ewing's sarcoma cells at mRNA and protein levels, including the transmembrane forms, which were also measured. After years of controversy due to the immune privilege of tumours expressing soluble Fas L, it has been shown recently that transmembrane Fas L is the form necessary for apoptosis induction (O’Reilly *et al*, 2009). The increased expression of a death receptor ligand, either alone or accompanied by the increase in its receptor, has been previously shown to induce cell death in Ewing's sarcoma cells. Abadie *et al* (2004) reported that a combination of cytokines (TNF-*α* with interferon *γ* or interferon *α* with interferon *γ*) induces cell death in SK-N-MC cells, and that this is mediated by the increased expression of TRAIL. A collaboration of TRAIL and interferon *γ* was also observed *in vivo* in a xenograft model of Ewing's sarcoma (Merchant *et al*, 2004). Similarly, inhibitors of metalloproteinases induce apoptosis in Ewing's sarcoma cell lines by avoiding the cleavage of transmembrane Fas L, thus increasing the level of transmembrane Fas L, as well as that of its receptor Fas (Mitsiades *et al*, 1999). In the present study, we also show that the rise in expression of Fas/Fas L by melatonin is responsible for the cell death induced by this molecule in SK-N-MC cells. This is supported by the finding of inhibition of melatonin-induced cell death by Fas- and Fas L-neutralising antibodies as well as by the lipooxigenase inhibitor NDGA, which has been associated with the inhibition of cell death mediated by Fas. There is abundant literature showing that the increase in death receptor expression potentiates cell death induced by the administration of the corresponding ligands. This is the particular case for melatonin, which sensitises glioma cells to TRAIL. Glioma cells are non-responsive to administration of TRAIL, whereas the antitumoural effect of melatonin when administered alone in glioma cells is due to its antiproliferative effect (Martin *et al*, 2006). However, melatonin increases the expression of the TRAIL receptor DR5, so the administration of TRAIL and melatonin in combination results in glioma cell death (Martin *et al*, 2010). Additionally, substances that increase expression of a death receptor and its ligand may induce cell death even when administered alone. This is the case of arachidonic acid in human leukaemia cells (Liu and Chang, 2009), apicidin in promyelocytic leukaemia cells (Kwon *et al*, 2002) or the inhibitors of metalloproteinases in Ewing's sarcoma cells; these substances are able to increase the expression of Fas, as well as Fas L, by avoiding transmembrane Fas L cleavage (Mitsiades *et al*, 1999). As already mentioned, treatment of Ewing's sarcoma cells with melatonin also has the ability to raise both Fas and Fas L (including transmembrane Fas L) expression, and so induce cytotoxicity.

It has been reported that there is an increase in free radicals in the apoptosis induced by the activation of death receptors and that antioxidants block such apoptosis (Lee *et al*, 2002; Devadas *et al*, 2003; Sato *et al*, 2004; Shen and Pervaiz, 2006). Furthermore, some pro-oxidant effects of melatonin have been recently reported in a few human leukaemia and hepatoma cell lines, where this molecule exerts cytotoxic effects (and only in these kinds of cells) (Osseni *et al*, 2000; Wolfler *et al*, 2001). In the present report, we also find an early and transient increase in intracellular oxidants, which is accompanied by an activation of the transcription factor NF-*κ*B. We show indeed that addition of antioxidants prevents the cytotoxicity of melatonin in Ewing's sarcoma cells, supporting the involvement of an increase in such oxidants in melatonin-induced cell death. Nuclear factor-*κ*B is one of the proteins directly regulated by the intracellular redox state, and it has been shown to be regulated by melatonin in other experimental systems (Chuang *et al*, 1996; Schoonbroodt and Piette, 2000). Nuclear factor-*κ*B activation is essential in activation-dependent Fas promoter induction (Chan *et al*, 1999). This role has been reported particularly in Fas expression induced by TNF-*α* (Starace *et al*, 2005). The fact that the addition of parthenolide, a well-known inhibitor of NF-*κ*B activation, prevents both Fas/Fas L regulation and induction of cell death by melatonin in these cells supports the implication of NF-*κ*B in melatonin cytotoxicity through the regulation of Fas/Fas L expression. The increase in oxidants, the preventive effect on cytotoxicity of antioxidants, the known role of free radicals in activating NF-*κ*B (Schoonbroodt and Piette, 2000) and the prevention of the increase in cell death and Fas/Fas L expression by the prevention of NF-*κ*B activation are in support of the implication of the rise in free radicals in the cytotoxic effect of melatonin in some particular types of tumours.

Melatonin also induced apoptotic cell death and upregulation of Fas/Fas L in the other Ewing's sarcoma cell lines studied. Induction of cell death by melatonin is something that is very uncommon. No cytotoxic effect of melatonin on normal cells has ever been reported, and it only induces cell death in a few cancer cell types (Trubiani *et al*, 2005; Buyukavci *et al*, 2006; Rubio *et al*, 2007; Martin-Renedo *et al*, 2008). In fact, its antitumoural properties are mostly mediated by antiproliferative effects (Hill and Blask, 1988; Sainz *et al*, 2003; Reiter, 2004; Martin *et al*, 2006). Fas/Fas L increase caused by melatonin corresponds specifically with cell death induction and is not a general effect of this molecule without consequences in other cell types; melatonin does not increase these proteins in other cell lines where it does not induce cell death. The fact that it only induces Fas/Fas L increase in the cells where it has cytotoxic effects emphasises that this is the mechanism involved and opens the possibility that the cytotoxic effects of melatonin in other cancer cell types may also be due to the regulation of death receptors and/or their ligands. In fact, we have recently published that melatonin induces cell death in human leukaemia cells in correlation with an increase in Fas and Fas L expression (Casado-Zapico *et al*, 2011).

The fact that melatonin only shows cytotoxicity in some cancer cell types makes it important to study its mechanisms of action to understand the differences between sensitive and non-sensitive cells, and could provide clues to address new therapeutic strategies in specific tumours. The mechanisms of the increase in Fas and Fas L expression by melatonin in these cancer cells and the implication of free radical increase as the trigger of the cytotoxic pathway deserve further attention and deeper study. Melatonin has been shown to be an antioxidant both *in vitro* and *in vivo*, making it important to find differences in the cell biology of sensitive tumours that could explain why it generates free radicals in these cells. Regulation of Fas/Fas L expression is limited to a few tumour cell types and gives a hypothetical explanation for the lack of reported relevant side effects of this molecule. Apoptosis induced by melatonin in Ewing's sarcoma cells by Fas/Fas L upregulation and its synergism with other chemotherapeutic agents offer new insights in the study of alternative therapeutic strategies in the treatment of this cancer.

## Figures and Tables

**Table 1:** Primers used for quantitative PCR

**Gene**	**Sequence 5′ → 3′**
TNFR1	Forward: 5′- TGCCTACCCCAGATTGAGAA -3′
	Reverse: 5′- ATTTCCCACAAACAATGGAGTAG -3′
	
TNF-*α*	Forward: 5′- ACAAGCCCAACAACAAGG -3′
	Reverse: 5′- ATCGGGATGCCAAAGAGG -3′
	
FAS-R	Forward: 5′- AGCTTGGTCTAGAGTGAAAA -3′
	Reverse: 5′- GAGGCAGAATCATGAGATAT - 3′
	
FAS-L	Forward: 5′- CACTTTGGGATTCTTTCCAT -3′
	Reverse: 5′- GTGAGTTGAGGAGCTACAGA -3′
	
DR4	Forward: 5′- CGATGTGGTCAGAGCTG-GTACAGC -3′
	Reverse: 5′- GGACACGGCAGAGCCTGTGCCATC -3′
	
DR5	Forward: 5′- GGGAGCCGCTCATGAGGAAGTTGG -3′
	Reverse: 5′- GGCAAGTCTCTCTCCCAGCGTCTC -3′
	
GAPDH	Forward: 5′- ATGGGGAAGGTGAAGGTCGG -3′
	Reverse: 5′- GACGGTGCCATGGAATTTGC -3′

Abbreviations: DR=death receptor; FAS-L=Fas ligand; FAS-R=Fas receptor; GAPDH=glyceraldehyde 3-phosphate dehydrogenase; TNF-*α*=tumour necrosis factor-*α*; TNFR1=tumour necrosis factor receptor-1.

## References

[bib1] Abadie A, Besancon F, Wietzerbin J (2004) Type I interferon and TNF*α* cooperate with type II interferon for TRAIL induction and triggering of apoptosis in SK-N-MC Ewing tumor cells. Oncogene 23: 4911–4920 15077162 10.1038/sj.onc.1207614

[bib2] Antolín I, Mayo JC, Sainz RM, del Brío MA, Herrera F, Martín V, Rodriguez C (2002) Protective effect of melatonin in a chronic experimental model of Parkinson's disease. Brain Res 943: 163–173 12101038 10.1016/s0006-8993(02)02551-9

[bib3] Bruck R, Aeed H, Avni Y, Shirin H, Matas Z, Shahmurov M (2004) Melatonin inhibits nuclear factor kappa B activation and oxidative stress and protects thioacetamide induced liver damage in rats. J Hepatol 40: 86–93 14672618 10.1016/s0168-8278(03)00504-x

[bib4] Buyukavci M, Ozdemir O, Buck S, Stout M, Ravindranath Y, Savasan S (2006) Melatonin cytotoxicity in human leukemia cells: relation with its pro-oxidant effect. Fundam Clin Pharmacol 20: 73–79 16448397 10.1111/j.1472-8206.2005.00389.x

[bib5] Casado-Zapico S, Martin V, García-Santos G, Rodriguez-Blanco J, Sánchez-Sánchez AM, Luño E, Antolin I, Rodriguez C (2011) Regulation of the expressión of death receptors and their ligands by melatonin in haematological cancer cell lines and in leukaemia cells from patients. J Pineal Res 50: 345–355 21392090 10.1111/j.1600-079X.2010.00850.x

[bib6] Casado-Zapico S, Rodriguez-Blanco J, García-Santos G, Martin V, Sánchez-Sánchez AM, Antolín I, Rodriguez C (2010) Synergistic antitumor effect of melatonin with several chemotherapeutic drug son human Swing sarcoma cancer cells: potentiation of the extrinsic apoptotic pathway. J Pineal Res 48: 72–80 20025643 10.1111/j.1600-079X.2009.00727.x

[bib7] Chan H, Bartos DP, Owen-Schaub LB (1999) Activation-dependent transcriptional regulation of the human Fas promoter requires NF-kappa B p50-65 recruitment. Mol Cell Biol 19: 2098–2108 10022897 10.1128/mcb.19.3.2098 PMC84003

[bib8] Chuang JI, Mohan N, Meltz ML, Reiter RJ (1996) Effect of melatonin on NF-kappa-B DNA-binding activity in the rat spleen. Cell Biol Int 20: 687–692 8969462 10.1006/cbir.1996.0091

[bib9] Damron TA, Ward WG, Stewart A (2007) Osteosarcoma, chondrosarcoma, and Ewing's's sarcoma: National Cancer Data Base Report. Clin Orthop Relat Res 459: 40–47 17414166 10.1097/BLO.0b013e318059b8c9

[bib10] de Alava E, Gerald WL (2000) Molecular biology of the Ewing's sarcoma/primitive neuroectodermal tumor family. J Clin Oncol 18: 204–213 10623711 10.1200/JCO.2000.18.1.204

[bib11] Devadas S, Hinshaw JA, Zaritskaya L, Williams MS (2003) Fas-stimulated generation of reactive oxygen species or exogenous oxidative stress sensitize cells to Fas-mediated apoptosis. Free Radical Biol Med 35: 648–661 12957657 10.1016/s0891-5849(03)00391-5

[bib12] Dignam JD, Lebovitz RM, Roeder RG (1983) Accurate transcription initiation by RNA polymerase II in a soluble extract from isolated mammalian nuclei. Nucleic Acids Res 11: 1475–1489 6828386 10.1093/nar/11.5.1475 PMC325809

[bib13] Friesen C, Herr I, Krammer PH, Debatin KM (1996) Involvement of the CD95 (APO-1/FAS) receptor/ligand system in drug-induced apoptosis in leukaemia cells. Nat Med 2: 574–577 8616718 10.1038/nm0596-574

[bib14] Fulda S, Debatin K-M (2006) Extrinsic vs intrinsic apoptosis pathways in anticancer chemotherapy. Oncogene 25: 4798–4811 16892092 10.1038/sj.onc.1209608

[bib15] Fulda S, Strauss G, Meyer E, Debatin KM (2000) Functional CD95 ligand and CD95 death-inducing signaling complex in activation-induced cell death and doxorubicin-induced apoptosis in leukemic T cells. Blood 95: 301–308 10607716

[bib16] Garcia-Santos G, Antolin I, Herrera F, Martin V, Rodriguez-Blanco J, Carrera MP, Rodriguez C (2006) Melatonin induces apoptosis in human neuroblastoma cancer cells. J Pineal Res 41: 130–135 16879318 10.1111/j.1600-079X.2006.00342.x

[bib17] Hayden MS, Ghosh S (2004) Signaling to NF-kappaB. Genes Dev 18: 2195–2224 15371334 10.1101/gad.1228704

[bib18] Herrera F, Martin V, García-Santos G, Rodríguez-Blanco J, Antolín I, Rodríguez C (2007) Melatonin prevents glutamate-induced oxytosis in the HT22 mouse hippocampal cell line through an antioxidant effect specifically targeting mitochondria. J Neurochem 100: 736–746 17263795 10.1111/j.1471-4159.2006.04228.x

[bib19] Hill SM, Blask DE (1988) Effects of the pineal hormone melatonin on the proliferation and morphological characteristics of human breast cancer cells (MCF-7) in culture. Cancer Res 48: 6121–6126 3167858

[bib20] Kontny HU, Hammerle K, Klein R, Shayan P, Mackall CL, Niemeyer CM (2001) Sensitivity of Ewing's sarcoma to TRAIL-induced apoptosis. Cell Death Differ 8: 506–514 11423911 10.1038/sj.cdd.4400836

[bib21] Kumar A, Jasmin A, Eby MT, Chaudhary PM (2001) Cytotoxicity of tumor necrosis factor related apoptosis-inducing ligand towards Ewing's's sarcoma cell lines. Oncogene 20: 1010–1014 11314037 10.1038/sj.onc.1204154

[bib22] Kwon SH, Ahn SH, Kim YK, Bae GU, Yoon JW, Hong S (2002) Apicidin, a histone deacetylase inhibitor, induces apoptosis and Fas/Fas ligand expression in human acute promyelocytic leukaemia cells. J Biol Chem 277: 2073–2080 11698395 10.1074/jbc.M106699200

[bib23] Lee MW, Park SC, Kim J-H, Kim I-K, Han KS, Kim KY, Lee WB, Jung Y-K, Kim SS (2002) The involvement of oxidative stress in tumor necrosis factor (TNF)-related apoptosis-inducing ligand (TRAIL)-induced apoptosis in HeLa cells. Cancer Lett 182: 75–82 12175526 10.1016/s0304-3835(02)00074-5

[bib24] Lissoni P, Barni S, Mandala M, Ardizzoia A, Paolorossi F, Vaghi M (1999) Decreased toxicity and increased efficacy of cancer chemotherapy using the pineal hormone melatonin in metastatic solid tumour patients with poor clinical status. Eur J Cancer 35: 1688–1692 10674014 10.1016/s0959-8049(99)00159-8

[bib25] Liu WH, Chang LS (2009) Arachidonic acid induces Fas and Fas L upregulation in human leukaemia U937 cells via Ca2+/ROS-mediated suppression of ERK/c-Fos pathway and activation of p38 MAPK/ATF-2 pathway. Toxicol Lett 191: 140–148 19720122 10.1016/j.toxlet.2009.08.016

[bib26] Martin V, García-santos G, Rodriguez-Blanco J, Casado-Zapico S, Sánchez-Sánchez AM, Antolin I, Medina M, Rodriguez C (2010) Melatonin sensitizes human malignant glioma cells against TRAIL-induced cell death. Cancer Lett 287: 216–223 19632770 10.1016/j.canlet.2009.06.016

[bib27] Martin V, Herrera F, Carrera-Gonzalez P, García-Santos G, Antolín I, Rodríguez-Blanco J, Rodriguez C (2006) Intracellular signaling pathways involved in the cell growth inhibition of glioma cells by melatonin. Cancer Res 66: 1081–1088 16424044 10.1158/0008-5472.CAN-05-2354

[bib28] Martin-Renedo J, Mauriz JL, Jorquera F, Ruiz-Andres O, Gonzalez P, Gonzalez-Gallego J (2008) Melatonin induces cell cycle arrest and apoptosis in hepatocarcinoma HepG2 cell line. J Pineal Res 45: 532–540 19012662 10.1111/j.1600-079X.2008.00641.x

[bib29] Merchant MS, Yang X, Melchionda F, Romero M, Klein R, Thiele CJ (2004) Interferon *γ* enhances the effectiveness of tumor necrosis factor-related apoptosis-inducing ligand receptor agonists in a xenograft model of Ewing's sarcoma. Cancer Res 64: 8349–8356 15548704 10.1158/0008-5472.CAN-04-1705

[bib30] Micheau O, Solary E, Hammann A, Martin F, Dimanche-Boitrel MT (1997) Sensitization of cancer cells treated with cytotoxic drugs to Fas-mediated cytotoxicity. J Natl Cancer Inst 89: 783–789 9182976 10.1093/jnci/89.11.783

[bib31] Mitsiades N, Poulaki V, Leone A, Tsokos M (1999) Fas-mediated apoptosis in Ewing's sarcoma cell lines by metalloproteinase inhibitors. J Natl Cancer Inst 91: 1678–1684 10511596 10.1093/jnci/91.19.1678

[bib32] Mitsiades N, Poulaki V, Mitsiades C, Tsokos M (2001) Ewing's sarcoma family tumors are sensitive to tumor necrosis factor-related apoptosis-inducing ligand and express death receptor 4 and death receptor 5. Cancer Res 61: 2704–2712 11289151

[bib33] O’Reilly LA, Tai L, Lee L, Kruse EA, Grabow S, Fairlie WD (2009) Membrane-bound but not secreted Fas Ligand is essential for Fas-induced apoptosis and prevention of autoimmunity and cancer. Nature 461: 659–663 19794494 10.1038/nature08402 PMC2785124

[bib34] Osseni RA, Rat P, Bogdan A, Warnet JM, Touitou Y (2000) Evidence of prooxidant and antioxidant action of melatonin on human liver cell line HepG2. Life Sci 68: 387–399 11205889 10.1016/s0024-3205(00)00955-3

[bib35] Picarda G, Lamoureux F, Geffroy L, Delepine P, Montier T, Laud K (2010) Preclinical evidence that use of TRAIL in Ewing's′s sarcoma and osteosarcoma therapy inhibits tumor growth, prevents osteolysis, and increases animal survival. Clin Cancer Res 16: 2363–2374 20371692 10.1158/1078-0432.CCR-09-1779

[bib36] Post A, Holsboer F, Behl C (1998) Induction of NF-kappaB activity during haloperidol-induced oxidative toxicity in clonal hippocampal cells: suppression of NF-kappaB and neuroprotection by antioxidants. J Neurosci 18: 8236–8246 9763469 10.1523/JNEUROSCI.18-20-08236.1998 PMC6792862

[bib37] Reis-Sobreiro M, Gajate C, Mollinedo F (2009) Involvement of mitochondria and recruitment of Fas/CD95 signaling in lipid rafts in resveratrol-mediated antimyeloma and antileukemia actions. Oncogene 28: 3221–3224 19561642 10.1038/onc.2009.183

[bib38] Reiter RJ (2004) Mechanism of cancer inhibition by melatonin. J Pineal Res 37: 213–214 15357667 10.1111/j.1600-079X.2004.00165.x

[bib39] Reiter RJ, Tan DX, Mayo JC, Sainz RM, Leon J, Czarnocki Z (2003) Melatonin as an antioxidant: biochemical mechanisms and pathophysiological implications in humans. Acta Biochim Pol 50: 1129–1146 14740000

[bib40] Rubio S, Estevez F, Cabrera J, Reiter RJ, Loro J, Quintana J (2007) Inhibition of proliferation and induction of apoptosis by melatonin in human myeloid HL-60 cells. J Pineal Res 42: 131–138 17286744 10.1111/j.1600-079X.2006.00392.x

[bib41] Sainz RM, Mayo JC, Rodriguez C, Tan DX, Lopez-Burillo S, Reiter RJ (2003) Melatonin and cell death: differential actions on apoptosis in normal and cancer cells. Cell Mol Life Sci 60: 1407–1426 12943228 10.1007/s00018-003-2319-1 PMC11138606

[bib42] Sato T, Machida T, Takahashi S, Iyama S, Sato Y, Kuribayashi K, Takada K, Oku T, Kawano Y, Okamoto T, Takimoto R, Matsunaga T, Takayama T, Takahashi M, Kato J, Niitsu Y (2004) Fas-mediated apoptosome formation is dependent on reactive oxygen species derived from mitochondrial permeability transition in Jurkat cells. J Immunol 173: 285–296 15210786 10.4049/jimmunol.173.1.285

[bib43] Schoonbroodt S, Piette J (2000) Oxidative stress interference with the nuclear factor-kappa B activation pathways. Biochem Pharmacol 60: 1075–1083 11007944 10.1016/s0006-2952(00)00371-3

[bib44] Shen MM, Pervaiz S (2006) TNF receptor superfamily-induced cell death: redox-dependent execution. FASEB J 20: 1589–1598 16873882 10.1096/fj.05-5603rev

[bib45] Starace D, Riccioli A, D’Alessio A, Giampietri C, Petrungaro S, Galli R (2005) Characterization of signaling pathways leading to Fas expresión induced by TNF-*α*: pivotal role of NF-*κ*B. FASEB J 19: 473–475 15601669 10.1096/fj.04-2726fje

[bib46] Trubiani O, Recchioni R, Moroni F, Pizzicannella J, Caputi S, Diprimio R (2005) Melatonin provokes cell death in human B-lymphoma cells by mitochondrial-dependent apoptotic pathway activation. J Pineal Res 39: 425–431 16207299 10.1111/j.1600-079X.2005.00270.x

[bib47] Uren A, Toretsky JA (2005) Ewing's's sarcoma oncoprotein EWS-FLI1: the perfect target without a therapeutic agent. Future Oncol 1: 521–528 16556028 10.2217/14796694.1.4.521

[bib48] Wolfler A, Caluba HC, Abuja PM, Dohr G, Schauenstein K, Liebmann PM (2001) Prooxidant activity of melatonin promotes fas-induced cell death in human leukemic Jurkat cells. FEBS Lett 502: 127–131 11583113 10.1016/s0014-5793(01)02680-1

